# Notes and News

**Published:** 1887-01-08

**Authors:** 


### Notes and News.

Collected and Communicated.

Our especial thanks are due to Lord Randolph Churchill, who has kindly consented to contribute an article to The Hospital, so soon as the political crisis is past. Lord Randolph has always displayed the deepest interest in the welfare of the people, and his utterance on this subject will no doubt excite much attention.

We have the pleasure to announce that Lord Brabazon, who has devoted his life to good deeds, and who, in consequence, seems likely to take the place in the affections of the people of the late Earl of Shaftesbury, will contribute his views to an early number, on the subject of the after-care and treatment of asylum inmates on their discharge from public institutions.

The Earl of Dalhousie has been elected President of the Brechin Infirmary during the ensuing year. Provost Lamb has been appointed Vice-President.

The Hospital Sunday Fund in Exeter this year has exceeded the amount of any previous year. In Hull, the collection shows a decrease of about £47; in Wolverhampton, one of £82.

The bazaar recently held in aid of the extension of St. Mary's Hospital, Manchester, has resulted in a sum of £420 being handed to the building fund.

Mr. William Fowler has sent a cheque for twenty-five guineas to the North London Hospital.

Professor Max Müller says that the hieroglyphic signs of our modern prescriptions have been traced back by Champollion to the hieroglyphics of Egypt.

The Worshipful Company of Sadlers have sent twenty guineas to the Charing Cross Hospital; ten guineas to the British Home for Incurables; and five guineas to the Metropolitan Convalescent Institution, at Walton-on-Thames. The Girdlers have sent ten guineas to the British Home for Incurables.

Although but four months have elapsed since the fund was started for extending the Sunderland Infirmary, it already amounts to over £5,000.

Sir Andrew Clark, Bart., M.D., F.R.S., has promised to preside at the 67th annual court of the Seamen's Hospital Society (late Dreadnought), to be held next February.

Mr. Duncan Caddy of The Hospitals Association has composed a spirited polka called "The Pearl," which he has dedicated to Miss Manson, Matron of St. Bartholomew's Hospital, the title being the sobriquet by which she was known when a probationer at the Manchester Royal Infirmary.

Dr. J. Aveling's "Mechanical Begging Boxes" at the late Colinderies proved a very successful experiment. The net proceeds which resulted from this ingenious mode of collection amounted to £404 5*s.* 8*d.*

As the Queen's Jubilee approaches, proposals, suggestions, and ideas come in, in bewildering numbers. Nearly everybody seems to have formulated a plan on which *he* thinks the great commemoration should be celebrated. One proposition which has been made is, that the *ladies* of Great Britain and Ireland, and the Colonies, should raise a fund for building in or near London a "Queen's Hospital" having a series of wards, each to be named after one of Her Majesty's children. and one for each Colony; while the *gentlemen* are to raise a second fund, the object of which shall be to pay off the debts now existing on our hospitals! Both schemes are admirable, but their proportions are, we fear, too gigantic to admit of realisation.

It is gratifying to find that all the principal provincial hospitals as well as the metropolitan ones are joining or sending representatives to the Hospitals Association, Norfolk House, Norfolk Street, Strand. The Board of Management of the Norfolk and Norwich Hospital is the latest instance; they have joined the Hospitals Association as members, and have requested to be supplied with The Hospital and other publications of the Association, as they may be issued from time to time. We dwell on the importance of co-operation in another column of the present issue, and we commend the example of the Norfolk and Norwich Hospital to the serious consideration of the managers of these institutions throughout the country.

The next meeting of the Hospitals Association will be held at the Outer Temple, Room 44, 225, Strand, (opposite the Royal Courts of Justice) on Wednesday the 19th inst., at 8 p.m., when Colonel Montefiore will read a paper on "Some Notes on Our Metropolitan Poor Law Infirmaries, and the Importance of Throwing Them Open to Medical Science." Dr. G. W. Potter, or Mr. William Adams, F.R.C.S., Chairman of the Highgate Infirmary, will take the chair, and a large attendance, with an interesting discussion of this important question, is anticipated. Cards of admission may be obtained on application to Mr. Morgan Jenkins, The Hospitals Association, Norfolk House, W.C.

Marvellous truly are the calculations which some writers make. Indeed, the performances of a Babbage or a Sol Stone sink into utter insignificance beside them. "Miranda" observed, in the *Lady's Pictorial*, the other day, "Every bed in every hospital the whole of the land through could be maintained in efficient comfort, and be attended with the highest medical and nursing skill, if only ten per cent. of the money spent upon granitic monuments and other churchyard statuary were given them." This reads admirably; but we would like to know, first, if "Miranda" ascertained the number of beds in every hospital throughout the "whole of the land"; and secondly, how she arrived at the sum total which people spend per annum upon "granitic monuments and other churchyard statuary."

STRANGE incidents crop up now and again in connection with hospitals. On Christmas Day, a labourer, named Hewett, living at Stanley Road, Fulham, was walking across his garden, when he stumbled and fell. It being discovered that he had fractured his right leg, the unfortunate man was at once removed to St. George's Hospital, where it was found necessary to perform amputation. Poor Hewett's run of ill-luck, in the shape of accidents, has been most remarkable, for he told one of the surgeons that this was the fourth Christmas Day in succession that he had passed within the walls of a hospital.

THE following vacancies are announced this week, the amount of the salary being placed in each case after the name of the institution :—

**Honorary Medical Officers.**

City of London Lying-In Hospital. Surgeon Accoucheur.

**Resident Medical Officers.**

Royal Infirmary, Ryde, I. W. House Surgeon, doubly qualified, and Secretary.—£50, with board, etc.
Worcester County Lunatic Asylum. Third Assistant, doubly qualified.—£100 with board, etc.
Northern Infirmary, Inverness. House Surgeon and Apothecary.—£50, with board, etc.
Newcastle-on-Tyne Dispensary. Doubly qualified.—£250, with furnished house.
North London Hospital for Consumption. Doubly qualified. —£40.
North West London Hospital. Assistant, doubly qualified.—Board and residence.
City of London Hospital for Diseases of the Chest. Qualified Clinical Assistant.—Six months, £20.

**Non-Resident Medical Officers.**

St. Asaph Union. Single qualification.—£78.

**Trained Nurses.**

North West London Hospital. Night.—£20, with uniform.
Northampton Nursing Institution. Several.—£25, with uniform.
Birmingham General Hospital. Two Surgical.—£25, with uniform.
Manchester Royal Eye Hospital. One.—£24, with uniform.
Netherfield Road Hospital, Liverpool Two Charge, £35. Three Assistant, £25, uniform, etc.
City of London Hospital for Diseases of the Chest. One Medical.—£20 to £24, with uniform, etc.
Burton-on-Trent Nursing Institution. One District, one Private.—£25 to £28, uniform, etc.
East Suffolk Hospital, Ipswich. Two.—£25 to £30, with board, uniform, etc.

MRS. MARY ANN GARSTON, late of Aigburth, near Liverpool, has left £100 to the Royal Infirmary, Liverpool.

DR. JOHN THEODORE CASH has been appointed to the professorship of Materia Medica in the University of Aberdeen, in the room of Dr. Dyce Davison, whose tragic death we recently recorded.

THE beds of the West London Hospital (which was recently closed for repairs) are rapidly filling up. About seventy of the 101 beds are already in use. The hospital has received £500 from the Homage Jury of the Manor of Fulham.

THE Camberwell Infirmary is about to be considerably enlarged. The present building, which accommodates only 230 persons, is always full, and large numbers of the sick poor of the parish have now to be received at the workhouse. The addition which it is proposed to make to the Infirmary will provide for about 400 extra patients.

THE Birmingham General Hospital has received £4,231 as its share in the recent hospital collection for that town. The total sum collected was £4,662. The balance, after deducting working expenses, has been divided among the smaller local charities.

THE good people of St. Albans are turning their attention to the sick poor of the town, and, in celebration of the approaching Jubilee, they have decided to sell the present wretched little building, and build a hospital moıe worthy of the town. The Corporation had another Jubilee scheme on hand, in the shape of a market, but they have now dropped this, and joined hands with the hospital governors.

THE Lord Mayor was greatly pleased with his recent visit to the Marylebone Infirmary. There is certainly no institution of the kind, within the metropolitan area, which can at all vie with it. This huge building, which has won the epithet of the "Model Infirmary," is capable of accommodating about 750 patients, but it has never yet been quite full. During the past year, about 2,500 cases were dealt with.

LONG service is a characteristic feature of many an old hospital official, from the house-governor down to the hall-porter, but the case of the late secretary of the Kent and Canterbury Hospital can, we should imagine, be paralleled by but very few others indeed. Mr. Thomas Soutee, who died only a few days since, was in the service of the hospital authorities for no less than fifty-two years. He retired not very long ago on a well-earned pension of £120 a year, supplemented by a sum of £260 which his friends in Canterbury and the neighbourhood collected for him.

THE new Fever Hospital for Newcastle, which is in course of erection at Walker, will, it is expected, be ready for the reception of patients about the middle of May next. The building was commenced about eighteen months ago by the Corporation of Newcastle, in consequence of the well-known inadequacy of the old hospital in Bath Lane to the demands made upon it, as well as from a feeling that the existing institution was, from its position in so crowded a neighbourhood, a possible source of danger and alarm. The new hospital is in the Queen Anne style, covers nearly ten acres of land, and will cost about £18,000. The site is surrounded by a brick wall, which will ensure privacy and quiet to the patients.

AT the first meeting of the Provisional Committee, appointed to consider the proposed erection of a hospital at Southend, in commemoration of the Queen's Jubilee, nearly £300 was subscribed in the room towards the advancement of the scheme. It is stated that such an institution has long been needed by the inhabitants of the wide district known as the Rochford Hundred, of which Southend may be considered the centre, and we hope they will follow the example so generously set by the members of the Committee, and that the proposed new institution will soon become an accomplished fact.

We have received several further letters, for two only of which we can find space, on the subject of "Sister Olive," and the right of ladies and others to marry who engage in hospital work. The letters, of course, express various views, and evidence a very proper feeling against any toleration being extended to flirtation in any form within the hospital walls. We have already expressed the opinion that any woman who enters a hospital for any other reason than a sincere love of the work by which she hopes to gain a livelihood, is not only unworthy, but unfit to act as a nurse. We believe that this is the general feeling, and that further discussion is neither desirable nor necessary.

A donation of £5, given by the Winsford Local Board to the Manchester Eye Hospital, has raised a curious quarrel. The Government auditor, in scrutinising the accounts of the Winsford magnates, took exception to the donation, on the ground that the Eye Hospital was not an institution for the reception of infectious or other cases for which the Local Sanitary Authorities are by law required to provide accommodation. The Local Government Board has, however, reversed the decision of the auditor, by remitting the surcharge on the Winsford Local Board made by this official, on the ground that the Public Health Act does not require the Local Board to provide hospital accomodation of any kind.

We have received the following account of how Christmas Day was spent from Miss M. J. Thompson, the matron of the Oldham Infirmary, and we very cordially reciprocate the good wishes of her staff and herself:

"Dear Mr. Editor,—We thought, perhaps, it might interest the readers of The Hospital to know how Christmas was passed in a provincial Lancashire Hospital. I send you the account of the Infirmary, cut from one of our local daily papers. On New Year's Eve the patients from the various wards were brought into the children's ward, where the Christmas-tree was dismantled. A number of ladies and gentlemen, who had kindly helped with subscriptions to the decoration fund, were present. Every patient (69) received a useful present in warm clothing from the Christmas-tree, besides other small presents. The tree, which was a very large one, was a source of great amusement to the children and visitors. After the Christmas-tree, a concert was given in another ward by the house-surgeon (Mr. F. Weaver) and nursing staff, assisted by two lady friends (one of the house-surgeon's sisters). It has been highly gratifying to us, to know and feel that our efforts this year have been appreciated by the patients, and also by the public, who have visited the wards in large numbers. One word more before closing, which is to say how highly myself and the nursing staff appreciate The Hospital, and the fact of our sending this report is from the interest we feel in your valuable paper. Wishing The Hospital a happy and prosperous New Year."

It is to be hoped that Her Majesty's Jubilee will find an abiding memorial at Westminster Hospital. It has been decided by the parochial authorities of St. Margaret's, in which parish this institution is situated, that the Queen's fiftieth year shall be celebrated in a two-fold character, one scheme being the endowment of a certain number of beds at this hospital, to which the sick children of the poor of this division of Westminster shall have special preference of admission. If the response to the appeal which has been made prove sufficiently liberal, it is proposed to endow a children's ward, to be styled the "St. Margaret's Jubilee Ward." The Baroness Burdett-Coutts, who is taking a warm interest in this project, has signified her intention to furnish a children's cot for Westminster Hospital in connection with this fund.

The tenth annual meeting of subscribers to the Adelaide Children's Hospital, South Australia, was held on the premises of the institution on Oct. 28th last. Mr. W. Howchin, the Secretary, read the report, from which it appeared that 294 in-patients, and 3,188 out-patients had been treated at the hospital during the year. The finances were stated to be in a thoroughly sound condition, and there was a good prospect that the hospital property (the Teetulpa Goldfields) would increase fifty per cent. in value during the next two years.

A suggestion has been made that Portsmouth should celebrate the Queen's Jubilee by the erection of a new hospital, to be called, of course, the "Victoria." The loyalty of a nation to a good sovereign is always pleasing to observe, but at the same time we should prefer to see a healthy discretion exercised by persons who, in the fulness of their benevolent intention, seek to build a hospital whenever the occasion offers. Portsmouth is not ill-provided for in respect to institutions for the relief of the indigent sick. To increase unwisely the number of our hospitals in the large centres of population means the still further impoverishment of the exchequers of existing institutions.

A correspondent draws our attention to the pernicious habit which visitors sometimes have, of attempting to convey surreptitiously to their friends in hospital such contraband articles as tobacco, brandy, etc. The only way of checking these well-meaning but ill-advised people is for sisters and nurses to exercise active vigilance on visiting days; but as sisters and nurses are not always Argus-eyed, it is simply impossible to check every infringement of regulations.

The Northern Counties Hospital for Incurables, though one of the youngest, is by no means one of the least, of the many noble charities which Lancashire boasts. During an existence of fifteen years the institution has been exceedingly fortunate in falling in with generous friends. Miss Kay, of Walmersley House, near Bury, has added her name to the list. A few months ago this lady gave Walmersley House, with its twenty-eight acres of land, as a free gift to the managers, who were desirous of extending the resources of their institution. The branch for the reception of forty female patients has since been opened, and now Miss Kay has further added to her munificence by defraying the expenses, amounting to £2,000, in connection with the alterations rendered necessary to adapt the House to the requirements of the poor sufferers.

